# Cranial suture lineage and contributions to repair of the mouse skull

**DOI:** 10.1242/dev.202116

**Published:** 2024-02-12

**Authors:** Daniel Doro, Annie Liu, Jia Shang Lau, Arun Kumar Rajendran, Christopher Healy, Marko Krstic, Agamemnon E. Grigoriadis, Sachiko Iseki, Karen J. Liu

**Affiliations:** ^1^ Centre for Craniofacial and Regenerative Biology, Faculty of Dentistry, Oral & Craniofacial Sciences, King's College London, London SE1 9RT, UK; ^2^ Department of Molecular Craniofacial Embryology and Oral Histology, Tokyo Medical and Dental University, 1-5-45 Yushima, Bunkyo-ku, Tokyo 113-8549, Japan

**Keywords:** Sutures, Stem cells, Cranial repair, Neural crest, Mesoderm, Bone

## Abstract

The cranial sutures are proposed to be a stem cell niche, harbouring skeletal stem cells that are directly involved in development, homeostasis and healing. Like the craniofacial bones, the sutures are formed from both mesoderm and neural crest. During cranial bone repair, neural crest cells have been proposed to be key players; however, neural crest contributions to adult sutures are not well defined, and the relative importance of suture proximity is unclear. Here, we use genetic approaches to re-examine the neural crest–mesoderm boundaries in the adult mouse skull. These are combined with calvarial wounding experiments suggesting that suture proximity improves the efficiency of cranial repair. Furthermore, we demonstrate that *Gli1*^+^ and *Axin2*^+^ skeletal stem cells are present in all calvarial sutures examined. We propose that the position of the defect determines the availability of neural crest-derived progenitors, which appear to be a key element in the repair of calvarial defects.

## Teaser

**Highlighted Article:** Examination of the embryonic origins of mouse cranial sutures by genetic labelling reveals the contributions of neural crest, mesoderm and skeletal stem cells, focusing on repair of calvarial wounds.

## INTRODUCTION

Craniofacial bone repair, which can be necessary as a result of accidents, congenital anomalies, or diseases such as cancer, is a daunting clinical challenge and a significant biomedical burden. Current treatment strategies include replacements; however, these do not truly mimic bone and can lead to difficulties in integration with other tissues, such as muscle. Poor healing can have severe impact on function and aesthetics, and multi-fragment breaks can be difficult to reconstruct (Lanza et al., 2020). Therefore, there is a need to improve our understanding of the endogenous craniofacial repair process, including a clearer definition of the osteogenic stem cell niche.

In craniofacial structures, the calvarial and facial bones form via intramembranous ossification, in which osteoblasts coalesce and differentiate directly within a membrane, without a cartilaginous intermediate (Nakashima and de Crombrugghe, 2003). Growth of these skull bones is organised at fibrous joints called sutures and occurs at the leading edges of the separate bones (Doro et al., 2017). Throughout embryonic and postnatal development, the sutures remain as active sites of bone formation. By early adulthood, when growth of the skull is complete, sutures become quiescent and gradually fuse. Nevertheless, cells residing in the sutures or adjacent periosteum retain the potential to heal calvarial bone in adults, following injury or disease (Doro et al., 2017). The healing capacity in the skull appears to rely on the sutural mesenchyme, which has recently been proposed to act as a stem cell niche (Zhao et al., 2015). In response to wounding, the skeletal mesenchyme rapidly undergoes proliferation, with sutural cells expanding toward the wound. Several groups have demonstrated that ablation of these resident skeletal stem cells blocks the healing capacity of the skull (Zhao et al., 2015; Maruyama et al., 2016; Wilk et al., 2017).

Here, we make use of a crucial and underappreciated observation: that adult frontal bones in the mouse heal more efficiently than parietal bones (Quarto et al., 2010; Doro et al., 2019). Studies from mouse models have demonstrated that the embryonic frontal bones are of neural crest origin, whereas the parietal bones are mesodermally derived (Jiang et al., 2002), raising the possibility that developmental history influences osteogenic potential. Indeed, we have demonstrated that adult neural crest-derived osteoblasts have an increased osteogenic capacity when cultured *in vitro* (Doro et al., 2019)*.* In contrast, parietal osteoblasts rarely generate osteogenic nodules (Doro et al., 2019); however, osteogenesis could be restored by co-culturing with neural crest-derived cells, either from frontal bones, or from the dura mater (Doro et al., 2019). Anecdotal evidence suggests a similarly improved healing capacity in human frontal bones (Skogh et al., 2013). These observations raise the possibility that there are increased numbers (or activity) of calvarial stem cells in sutures adjacent to the frontal bones. Most of the original studies comparing frontal and parietal healing efficiency do not account for the role of suture-derived osteoprogenitors, or the relevance of defect position in relation to the sutures. More recently, it has been demonstrated that bone healing is not an evenly distributed event across the parietal bone surface (Park et al., 2016). Although these studies show a correlation between repair efficiency and suture proximity, they disregard the dual embryonic origin of the cranial sutures and the possibility that distinct lineage contributions may influence repair. Furthermore, several studies suggest that neural crest-derived cells, and not the mesoderm, play key roles in the pathogenesis of craniofacial malformations (Hari et al., 2002; Brault et al., 2001; Wu et al., 2017), but also that neural crest may account for differences in cranial healing efficiency upon injuries of every sort (Quarto et al., 2010; Li et al., 2010; Senarath-Yapa et al., 2013).

Based on historical lineage-tracing assays using *Wnt1::cre*-driven ROSA26-*lacZ* reporters (Jiang et al., 2002; Wu et al., 2017; Yoshida et al., 2008), both interfrontal and sagittal sutures have been shown to be neural crest-derived, whereas the coronal suture is mesodermal. These studies focused on late embryonic/early post-natal stages, when cranial sutures are not yet clearly defined. To date, no definitive description of the neural crest–mesoderm boundary in the murine cranial vault has been established. Our overall hypothesis is that, in addition to suture proximity, the embryonic origins of the cranial sutures are also relevant when assessing repair. We hypothesise that calvarial sutures, notably those with majority neural crest contributions, may harbour more substantial skeletal stem cell populations. Thus, for non-neural-crest-derived bone, such as the parietal bones, proximity to specific sutures is crucial for homeostasis as well as cranial repair.

In this study, we use genetic labelling to compare *Wnt1::cre^+^* and *Mesp1::cre^+^* lineages, which define the proposed neural crest-mesoderm domains of the calvarium. We confirm that murine frontal bones heal more efficiently than parietal bones and note that the position of the defect in relation to the sutures is paramount for the outcome. We then demonstrate that *Wnt1::cre^+^* cells are found in every subcritical defect regardless of the embryonic origin of the wounded bone, or the proximity to a neural crest suture. We find the presence of *Axin2^+^* and *Gli1^+^* stem cell populations therein, noting the contributions of *Gli1*-*CreER^T2^*-positive cells in the healing wounds. This suggests that localised sources of neural crest cells and skeletal stem cells play a crucial role in healing of mesodermal skull bones.

## RESULTS AND DISCUSSION

### Neural crest-mesoderm boundary separates cranial sutures into distinct domains

In mouse, *Wnt1::*cre-dependent lineage labelling has demonstrated the neural crest-origin of the frontal bones whereas *Mesp1::cre-*labelled mesoderm contributes to the parietal bones (Jiang et al., 2002; Wu et al., 2017; Yoshida et al., 2008). However, the embryonic origins of the cranial sutures are debatable, as they rely on early observations that lacked resolution. Nevertheless, we and others have repeatedly confirmed a higher osteogenic potential of neural crest osteoblasts compared with mesoderm osteoblasts (Quarto et al., 2010; Doro et al., 2019; Behr et al., 2010a; Li et al., 2013). We sought to determine the neural crest-mesoderm domain and the presence of stem cell lineages in calvarial sutures using high-resolution lineage tracing.

**Fig. 1.**
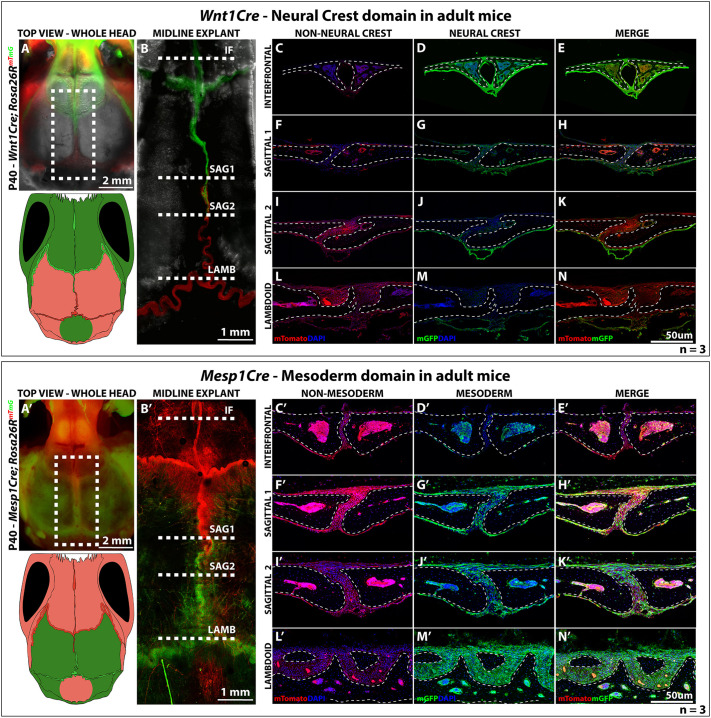
**Embryonic origins of the cranial sutures.** (A) Top view of P40 *Wnt1Cre; Rosa26R*^mTmG^mouse. Grey, brightfield; green, neural crest; red, non-neural-crest tissue. Dashed box shows area of the midline explant in B. Scale bar: 2 mm. Schematic depicts the neural crest-mesoderm domain in the cranial vault based on the linage tracing shown in A. (B) Confocal scan of the midline explant confirming the neural crest–mesoderm boundary at the middle of the sagittal suture. Scale bar: 1 mm. (C-N) Confocal scans of coronal sections at different regions of the midline showing non-neural-crest tissue (C,F,I,L; red), neural-crest tissues (D,G,J,M; green) and merged channels (E,H,K,N). Nuclear staining is shown in blue. Dashed lines in B show estimated planes of section in C-N. IF, interfrontal suture (C-E), SAG1, sagittal suture region 1 (F-H), SAG2, sagittal suture region 2 (I-K), LAMB, lambdoid suture (L-N). Scale bar: 50 µm. *n*=3. (A′) Top view of P40 *Mesp1::cre; Rosa26R*^mTmG^mouse. Green, mesoderm; red, non-mesodermal tissue. Dashed box shows area of the midline explant in B′. Scale bar: 2 mm. Schematic depicts the neural crest-mesoderm domain in the cranial vault based on the linage tracing shown in A′. (B′) Confocal scan of the midline explant confirming the neural crest–mesoderm boundary at the middle of the sagittal suture. Scale bar: 1 mm. (C′-N′) Confocal scans of coronal sections at different regions of the midline showing non-mesodermal tissue (C′,F′,I′,L′; red), mesodermal tissues (D′,G′,J′,M′; green) and merged channels (E′,H′,K′,N′). Nuclear staining is shown in blue. Dashed lines in B′ show estimated planes of section in C′-N′ (abbreviations as in B-N). Scale bar: 50 µm. *n*=3. Dashed lines in C-N,C′-N′ outline bone.

Transgenic *Wnt1::cre* mice were crossed with mice carrying a Cre-responsive *Rosa26R^mTmG^* reporter. In these mice, *Wnt1::cre* expression begins approximately at embryonic day (E) 8.5 in the dorsal neural tube, just as cranial neural crest cells are induced (Danielian et al., 1998). Soon after, these cells migrate into the cranial vault and can be tracked with membrane green fluorescent protein (mGFP). Cells lacking *Wnt1::cre* are labelled with membrane tomato (mTom) and are presumptive mesoderm. As expected, at postnatal day (P) 40, frontal bone shows exclusive neural crest contribution (mGFP^+^) as opposed to the parietal bones, which are of mesodermal origin, lacking mGFP (Fig. 1A). When we examined the midline sutures, we observed a clearly defined neural crest–mesoderm boundary, which lies right at the middle of the sagittal suture, delimiting a neural crest domain (interfrontal, coronal and anterior-sagittal suture) and a mesoderm domain (posterior-sagittal and lambdoid suture) (Fig. 1A,B). A coronal section at a more anterior part of the sagittal suture shows complete absence of mesoderm (Fig. 1B,C-E), contrasting a more posterior section, which shows very few neural crest cells with abundant mesoderm (Fig. 1F-K). Likewise, the interfrontal suture exhibited only neural crest (Fig. 1C-E), whereas the lambdoid suture showed only mesoderm (Fig. 1B,L-N). Altogether, this shows that the sagittal suture has dual embryonic origin with a well-defined neural crest–mesoderm boundary that extends past the anterior edge of the parietal bones, recapitulating the pattern observed in early development. This separates the sutures into two domains: the neural crest-derived sutures (interfrontal, coronal, squamous and anterior sagittal) and the mesodermal sutures (lambdoid and posterior sagittal).

We then performed the parallel experiment with *Mesp1::cre* males bred with females carrying a Cre-responsive *Rosa26R^mTmG^* reporter (Saga et al., 1999; Muzumdar et al., 2007). The *Mesp1::cre* transgenic animals carry Cre recombinase under the control of the endogenous *Mesp1* promoter, which activates initially a gastrulation, and later cranial mesenchyme (Yoshida et al., 2008). Yoshida and colleagues used *Mesp1::cre* animals to demonstrate the mesodermal contributions to the calvarial skeleton. We were able to confirm the putative neural crest–mesoderm boundary in mouse skulls at P40 (Fig. 1). *Mesp1::cre; Rosa26R^mTmG^* mice clearly showed boundaries at the midpoint of the sagittal suture (Fig. 1B′,F′-K′). We observed no positive labelling within the most rostral domains (Fig. 1C′-E′) adjacent to the interfrontal suture, except the minimal marrow cavity. The posterior sections at the lamboid suture (Fig. 1L′-N′) were entirely positive for *Mesp1::cre*. Of note, using this approach we did see both Mesp1::cre-positive and -negative cells in the rostral part of the sagittal suture (‘SAG1’; Fig. 1F′-H′), consistent with this domain having dual contributions from neural crest and mesoderm.

### Suture proximity determines the outcome of a subcritical cranial defect

**Fig. 2.**
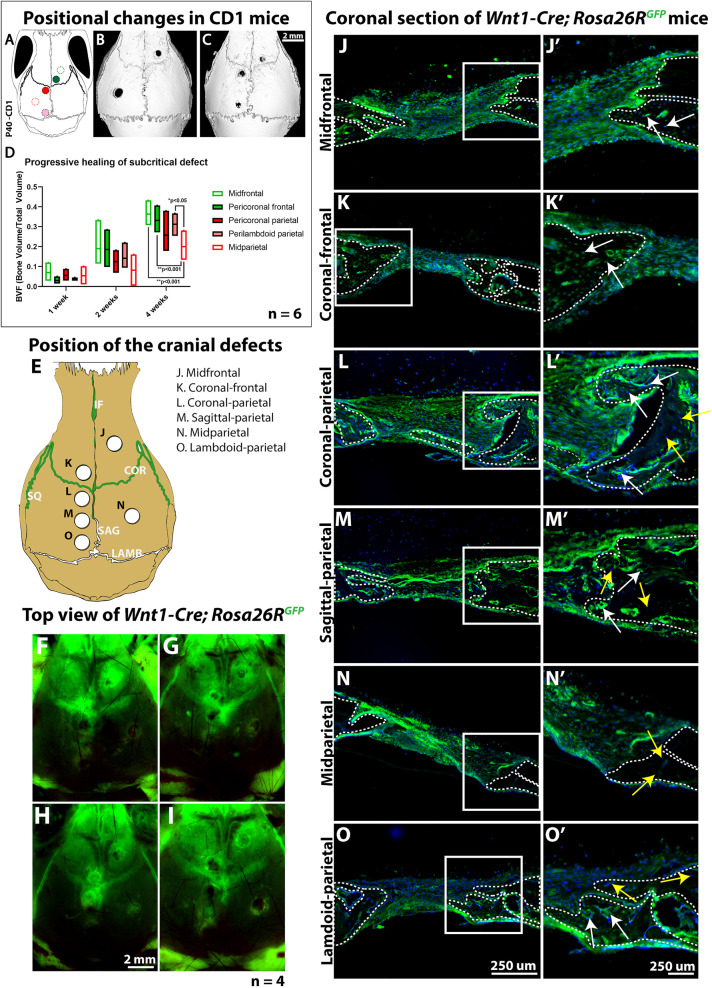
**Subcritical repair efficiency correlates with suture proximity irrespective of the calvarial bone, and neural crest cells are recruited to every defect.** (A) Schematic showing the position of cranial defects in relation to adjacent sutures. Green outline, midfrontal; solid green, peri-coronal frontal; solid red, peri-coronal parietal; red outline, midparietal; solid pink, peri-lambdoid. (B,C) Top view microCT scan of CD1 mouse heads 4 weeks after 1 mm subcritical defect surgery. (B) Defects equidistant from surrounding sutures in right frontal and left parietal bones. (C) Defects proximal to coronal and interfrontal suture (right), coronal and sagittal suture (top left), and sagittal and lambdoid suture (bottom left). (D) Bone volume fraction (BVF) analysis over a 4-week period post-subcritical defect surgery. Lower and upper box limits indicate the lower and upper quartile of BVF values, respectively. Horizontal line indicates the median. Upper and lower whiskers indicate minimum and maximum BVF values. *n*=6. *P<0.05, **P<0.001 (two-tailed, unpaired *t*-test). (E) Schematic showing the position of six subcritical defects (1 mm). Sutures are indicated in the picture as follows: COR, coronal suture; IF, interfrontal suture; SQ, squamous suture; SAG, sagittal suture; LAMB, lambdoid suture. (F-I) Top view of 40-day-old *Wnt1-Cre; Rosa26R^GFP^* mice 1 week after the surgical procedure. Green staining indicates neural crest-derived tissue. (J-O′) Coronal sections at the centre of each defect described in E. Neural crest-derived cells are shown in green, nuclear staining in blue. Dashed lines outline the bones, and the boxed area is shown at high magnification in J′-O′. Yellow arrowheads indicate non-neural crest-derived cells, white arrowheads neural crest-derived cells. *n*=4.

Although regional differences in repair efficiency have previously been reported, the dura mater and periosteum were thought to be the main sources contributing osteoprogenitors for cranial repair (Ochareon and Herring, 2011; Greenwald et al., 2000). Here, we set out to determine the role of proximity to the sutural niche. Subcritical defects (termed midfrontal and midparietal) of 1 mm width were made in the frontal bone of adult P40 mice, equidistant to the interfrontal and coronal sutures, as well as in the parietal bone, equidistant to the lambdoid, squamous, sagittal and coronal sutures (Fig. 2A, unfilled, dotted outlines; 2B). After 4 weeks, we observed a striking difference between midfrontal and midparietal repair (Fig. 2D), which accords with previous observations (Quarto et al., 2010; Li et al., 2010; Senarath-Yapa et al., 2013; Behr et al., 2010b). When the defects were made proximally to the sutures (peri-coronal frontal, peri-coronal parietal and peri-lambdoid) (Fig. 2A, filled, dotted outlines; Fig. 2C), no significant difference in repair was seen between frontal and parietal bones (Fig. 2D), whereas the peri-coronal parietal defect healing was comparable to that of the frontal bones (Fig. 2D). Our findings corroborate recent studies that show that subcritical parietal defects are more likely to heal the closer they are to the cranial sutures (Park et al., 2016). However, we did not see any specific increase in repair in the vicinity of a specific suture in relation to the other. This shows that, even though the sutures may contribute differently to cranial repair, the proximity to any suture is enough to provide subcritical healing, whereas the more distant parietal defect (here termed midparietal) seems to exceed the maximum critical distance from a suture.

### Neural crest lineage contributes progenitors to any cranial defect

We then assessed the extent to which *Wnt1::cre-*expressing neural crest cells infiltrate the repair site after injury. Subcritical defects were made at six distinct locations in *Wnt1::cre; Rosa26R*^+/mTmG^ mice (Fig. 2E). Defects varied from midfrontal (J) and coronal-frontal (K), which are surrounded by neural crest-derived interfrontal and coronal sutures, to lambdoid-parietal (O), which is only surrounded by mesoderm-derived sutures. One week after craniotomy, *Wnt1::cre^+^* cells were detected around and/or within all defects (Fig. 2F-I). This was clearly observed in coronal sections (Fig. 2J-O′). Even the defects in bones that are mesodermal in origin were filled with a large proportion of mGFP^+^ cells, suggesting the infiltration of neural crest-derived progenitors (Fig. 2M-O′). Importantly, within the frontal bone outlines, every cell was expected to be GFP^+^ (white arrows) as these bones are of neural crest origin (Fig. 2J′,L′). In contrast, within parietal bone outlines we observed a mix of GFP^−^ (yellow arrows) and GFP^+^ (white arrows) cells (Fig. 2L′,M′,O′), suggesting that new bone was formed from neural crest-derived progenitors, given that parietal bones are expected to be mesodermal. One exception is the midparietal defect (Fig. 2N′), which maintained its original flat edges, as obtained by the cylindrical nature of the drill bit, indicating minimal repair. This wound had an absence of green cells within the bone outline. This is consistent with previous observations of poor healing capacity of the mid-parietal bone region.

Although the osteogenic potential of neural crest versus mesoderm sutures has not yet been defined, it is intriguing that *Wnt1::cre*^+^ neural crest cells seem to contribute to the repair of every defect, including those in a mesoderm-dominant area (Fig. 2). Although we propose that the suture proximity is a key factor in repair capacity, it is also worth noting that the underlying dura mater is also neural crest derived, and a recent study has shown contributions of dura mater cells to suture regeneration (Yu et al., 2021). Regardless, neural crest-derived cells appear to be required for efficient repair, supporting previous observations that neural crest osteoprogenitors have higher osteogenic capabilities than other mesodermally derived calvarial cell populations.

### Stem cell populations are present in every calvarial suture and contribute to calvarial repair

**Fig. 3.**
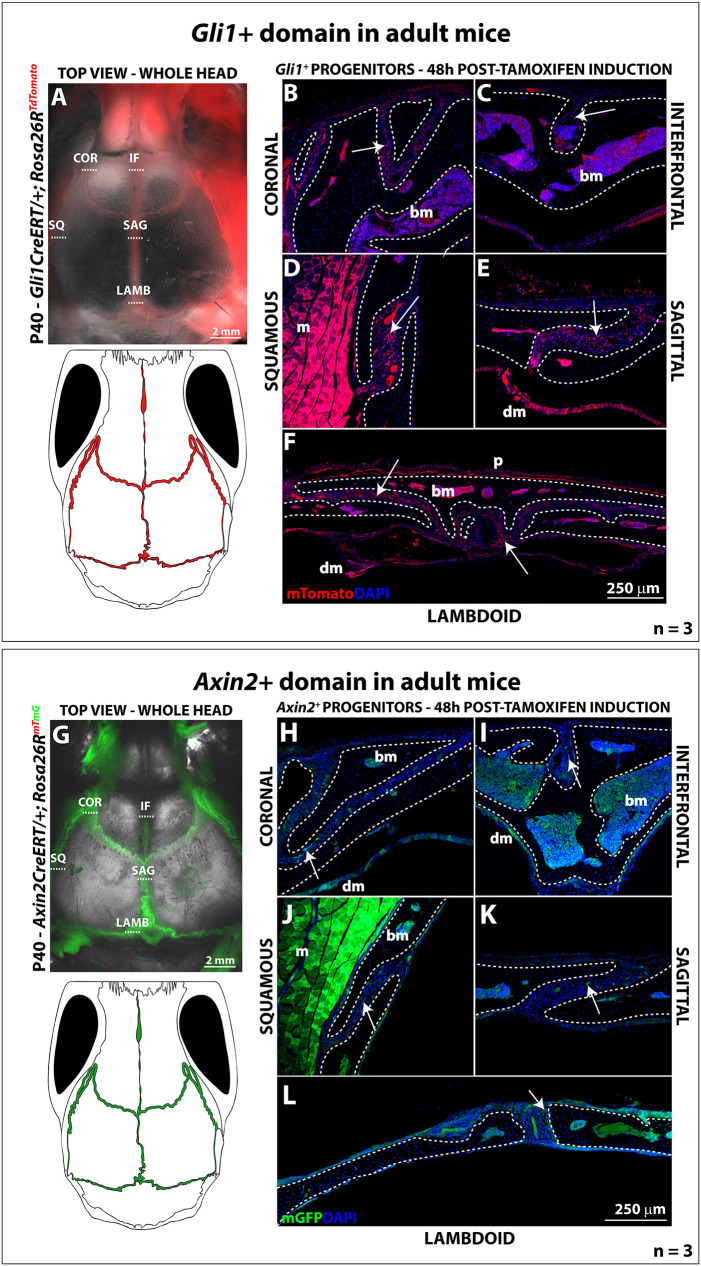
***Gli1^+^*-derived and *Axin2^+^*-derived progenitors are found in all cranial sutures.** (A) Top view of 40-day-old *Gli1CreERT*/+*; Rosa26R*^TdTomato^ mouse 48 h after tamoxifen induction. Grey, brightfield; red, *Gli1^+^* domain. Red staining on the right side is autofluorescence of opaque tissue remaining in the sample. COR, coronal suture; IF, interfrontal suture; SQ, squamous suture; SAG, sagittal suture; LAMB, lambdoid suture. Scale bar: 2 mm. Schematics show the *Gli1^+^* domain in the transgenic mouse calvarium. (B-F) Coronal sections of the calvarial sutures at the regions marked in A. Dashed lines show the bone outline. White arrows show red *Gli1^+^* cells. Scale bar: 250 µm. *n*=3. (G) Top view of 40-day-old *Axin2CreERT*/+*; Rosa26R^mTmG^*mouse 48 h after tamoxifen induction (red channel not shown). Grey, brightfield; green, *Axin2^+^* domain. Abbreviations as in A-F. Scale bar: 2 mm. Schematics show the *Axin2^+^* domain in the transgenic mouse calvarium. (H-L) Coronal sections of the calvarial sutures at the regions marked in G. Dashed lines show the bone outline. *Axin2^+^* cells are in green. White arrows show green *Axin2^+^* cells. bone marrow (bm), muscle (m), dura mater (dm) and periosteum (p) are notoriously autofluorescent tissues. Scale bar: 250 µm.

Several putative calvarial stem cell markers have been identified: the Hedgehog-pathway transcription factor *Gli1* (Zhao et al., 2015)*,* the Wnt-responsive gene *Axin2* (Maruyama et al., 2016) and the transcription factor *Prx1* (*Prrx1*) (Wilk et al., 2017)*.* Here, we investigate the presence of *Gli1^+^* and *Axin2^+^* stem cell populations using *Gli1::creER^T2^* or *Axin2::creER* drivers in combination with *Rosa26*^Tomato^ and *Rosa26R*^mTmG^ reporters, respectively. To label cells derived from *Gli1*^*+*^ and *Axin2*^+^ populations, 38-day-old mice were given tamoxifen and the heads were collected 2 days after injection. Top view and coronal sections of *Gli1*-*CreER^T2^; Rosa26*^Tomato/+^ heads revealed the abundant presence of *Gli1^+^* cells in all calvarial sutures (Fig. 3A-F). The analogous experiment with *Axin2-CreER^T2^; Rosa26R*^+/mTmG^ animals revealed the presence of *Axin2^+^-*derived cells also in every suture; however, they were sparse in comparison with *Gli1^+^* (compare Fig. 3G-L with 3A-F). Overall, we observed no apparent difference in *Gli1^+^* and *Axin2^+^* cell content in the cranial sutures examined. These findings are consistent with prior proposals that the *Axin2^+^* cells could be a subset of *Gli1^+^* progenitors (Maruyama et al., 2016; Wilk et al., 2017). Further assessment of *Axin2* mRNA expression on the *Gli1^+^-*derived cells would be necessary to define definitively these subpopulations of skeletal stem cells.

**Fig. 4.**
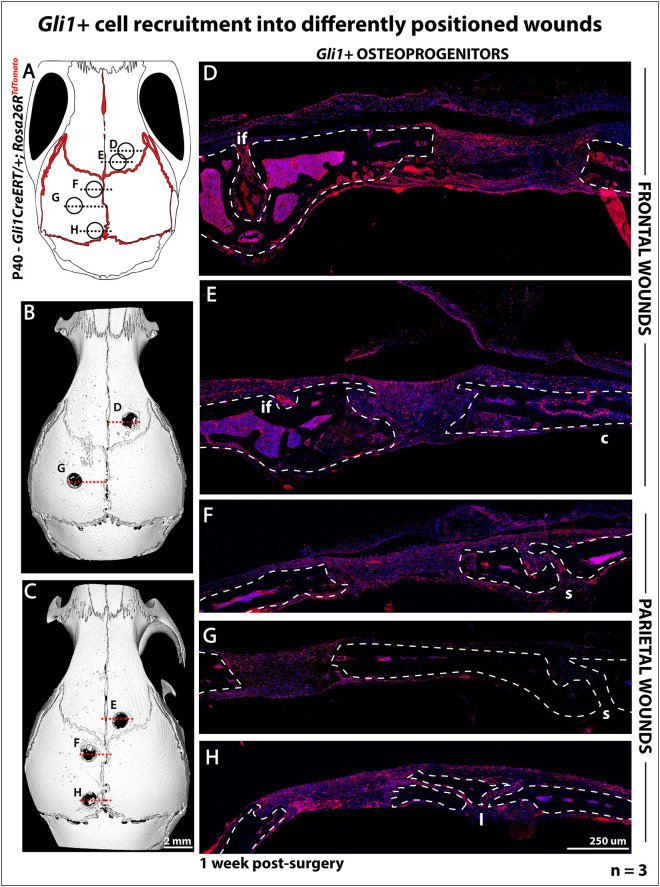
***Gli1^+^-*derived cells are found in every subcritical defect after 1 week.** (A) Schematics show sites of 1 mm subcritical defects in P40 *Gli1CreERT*/+*; Rosa26R*^TdTomato^mice. Circular outlines mark the positions of the wounds in relation to the different bones and sutures. (B,C) Top-view CT scans of heads after 1 week from the surgical procedure. Red dashed lines correspond to the approximate plane of sections in D-H. (D-H) Confocal scans of coronal sections at different wound sites 1-week post-surgery. Red, *Gli1^+^* cells. Dashed lines outline bone. if, interfrontal suture; c, coronal suture; s, sagittal suture; l, lambdoid suture. Scale bar: 250 µm. *n*=3.

To investigate whether *Gli1^+^* osteoprogenitors could populate all healing defects, we labelled *Gli1::creER^T2^; Rosa26*^Tomato^ mice as described. Subcritical defects were made at five sites of *Gli1*-*CreER^T2^; Rosa26*^Tomato/+^ mice as indicated (Fig. 4A) and *Gli1^+^* cells were examined in healing wounds 1-week post-surgery. Coronal sections of the wounds shown in Fig. 4B,C reveal that *Gli1^+^* cells (in red) were present in all wounds (Fig. 4D-H). Cells were present regardless of whether wounds were made in neural crest-derived frontal bone (Fig. 4D,E) or parietal (Fig. 4G,H). In the parietal bones, *Gli1^+^* cells were also seen regardless of proximity to the sagittal suture (Fig. 4G, compared with Fig. 4F,H). This suggests that, although midparietal defects (Fig. 4G) were substantially more distant to the sutures than the other defects were, they were still supplied with suture-derived osteoprogenitors, revealing the reparative capability of the suture mesenchyme in relation to distant defects. In the future, a quantitative assessment of the signalling events and cell content will be important reveal the mechanistic links between distance, cell identity and healing.

### Conclusion

Our data suggest that the embryonic origins of the cranial bone may predict the outcome of defect repair based on the neural crest composition of the intervening sutures and the presence of suture-residing osteoprogenitors relative to the calvarial wound site. However, it is important to note that our observations are based on several crucial assumptions. First, the genetic approaches used in our studies, although powerful, cannot exclude the possibility that these transgenic lines are not reactivated later in development, or subject to unknown transcriptional influences, or are simply not Cre responsive. Second, it is important to note that meninges, notably the dura mater, are also neural crest derived (Yoshida et al., 2008; Gagan et al., 2007; Dasgupta and Jeong, 2019). Indeed, we and others have demonstrated that the dura mater has osteogenic capacity (Petrie et al., 2008; Peptan et al., 2007), and when co-cultured with parietal cells are capable of nucleating osteogenesis (Doro et al., 2019). Finally, the composition of the sutural mesenchyme is surely dynamic, as the biological requirements change from embryogenesis to adult aging. Cell migration and mixing in adult life has been reported to occur in the sutures and in the meninges (Gagan et al., 2007; Deckelbaum et al., 2012). Furthermore, the meninges, which are comprised of neural crest cells during development, seem later to be invaded by mesodermal derivatives, leaving the remaining neural crest cells to act as resident stem cells in later life.

In the future, it will be important to track more localised cell contributions, such as dura mater-derived cells or the adjacent periosteal cells. It will also be important to assess the environment unique to the frontal versus parietal bones: for example, Marghoub and colleagues have demonstrated distinct mechanical forces in the anterior versus the posterior sutures, owing to differences in bone size, anatomy and underlying brain morphology (Marghoub et al., 2018). Altogether, although we cannot definitively state that availability of neural crest cells is the key element, we can nevertheless conclude that lineage identity and spatial positioning are both important in relation to the healing of calvarial defects.

## MATERIALS AND METHODS

### Animal procedures

All procedures were approved by King's College London ethical review process and performed in accordance with UK Home Office guidelines Project Licence P8D5E2773 (K.J.L.) or by the Institutional Animal Care and Use Committee of Tokyo Medical and Dental University [A2019-060C3 (S.I.)].

Mouse lines used were CD-1 mice (obtained from Charles River Laboratories), and *Rosa26R^mTmG^* (MGI ID 3716464), *Rosa26R^TdTomato^* (MGI ID 3809523), *Rosa26R-eGFP* (MGI ID 2136519) mouse reporter lines (all described previously: Muzumdar et al., 2007; Madisen et al., 2010; Mao et al., 2001). The following Cre drivers were used: *Wnt1::cre* (MGI ID 2386570), *Axin2::CreERT2* (MGI ID 5433373), *Gli1::CreERT2* (MGI ID 3053957), *Mesp1::cre* (MGI ID 2176467) (Danielian et al., 1998; Saga et al., 1999; van Amerongen et al., 2012; Ahn and Joyner, 2004).

### Subcritical defects

P40 mice were weighed and anaesthetised with an appropriate dose (10 μl/g of body weight) of a 10 mg/ml ketamine/2 mg/ml xylazine cocktail (Vetalar^®^, Zoetis; Rompun^®^, Dechra). The state of deep anaesthesia was confirmed through tail flick test and hind paw withdrawal response. Once the animals were heavily sedated, the fur on the top of the head was shaved using a hair trimmer. A sagittal incision was performed along the midline with a scalpel. The periosteal layer was then removed with the help of a cotton bud and 1-mm-width defects were drilled at top speed (50,000 rpm) into frontal and parietal bones of the mouse skull using a dental hand drill (Handy-ECO 1000, Marathon^®^). The defects were carefully performed to avoid injuring the dura mater. The bone surface was then rinsed with sterile PBS to remove any debris and the sagittal incision was sutured with 6-0 ETHILON^®^ nylon absorbable suture. Finally, the mice were moved to a 28°C incubator until full recovery. For the CD1 mice shown in Fig. 2, six mice received two wounds distant from the sutures and six mice received three wounds proximal to the sutures. *Wnt1-Cre; Rosa 26R^GFP^* mice (*n*=4) all received six wounds each, according to the schematics. The *Gli1CreERT; Rosa26R^tdTomato^* shown in Fig. 4 (*n*=3) received separate wounds similarly to CD1 in Fig. 2, i.e. three animals with two wounds and the other three with three wounds.

### Tamoxifen injection

Cre induction in *Cre^ERT^* mice was performed by peritoneal injection of a 10 mg/ml tamoxifen solution (Sigma-Aldrich) in the adult mouse at the desired stage. The solution was previously prepared by dissolving 10 mg of tamoxifen into 100 μl of absolute ethanol and 900 μl of corn oil. The dosage was determined according to the following: 1.5 mM/g of body weight in a volume of 7.5 μl/g of body weight.

### Micro-CT scanning

All head samples were fixed for 48 h at room temperature in 4% paraformaldehyde and scanned using a Scanco Medical µCT50^®^ with the following settings: energy 70 kV, intensity 114 µA, resolution 10 µm/voxel. The images were then reconstructed on Parallax Microview^®^ with isosurface image threshold set to 6000 and surface quality factor set to 40% with decimation factor of 0%.

To determine the bone volume fraction of each subcritical defect, a cylindrical region of interest calibrated to the volume of a 1 mm defect was used as total volume (mm^3^) and bone volume (mm^3^) was then measured using the Bone Analysis Tool on MicroView^®^.

### Sample fixation and sectioning

Samples were fixed in 4% paraformaldehyde for 48 h at 4°C. After three PBS washes, the skull cap was dissected and decalcified in 10% formic acid. After three more PBS washes, the samples were moved to a 30% sucrose solution in PBS until they sunk. The embedding solution was then replaced with a 30% sucrose solution mixed (1:1) with OCT compound (CellPath^®^). The samples were incubated at 4°C for another 48 h. Finally, the samples were moved and oriented in a plastic Tissue-Tek^®^ Cryomold^®^ filled with OCT compound. The mould was quickly moved into a dry-ice bath with absolute ethanol until the OCT block was fully solidified. Cryosections of 15 μm thickness were taken using OFT5000^®^ cryostat microtome (Bright Instruments^®^) and mounted onto Superfrost Ultra Plus^®^ slides (Thermo Fisher Scientific^®^), which were then stored at −80°C for future use.

### Immunostaining

GFP and RFP (Tomato) fluorescence in all experiments was assessed by immunofluorescence staining. Primary antibodies were: anti-GFP (ab13970, Abcam^®^), anti-RFP (5F*, ChromoTek^®^). Secondary antibodies were: Alexa Fluor™ 488 goat anti-chicken (A-11039, Invitrogen^®^), Alexa Fluor™ 568 goat anti-rat (A-11077, Invitrogen^®^). After dissolution of the OCT in PBS, slides were blocked with 1% bovine serum albumin (Sigma-Aldrich^®^), 10% goat serum (Gibco^®^), 0.05% Tween 20 (Sigma-Aldrich^®^) in PBS. Primary antibodies were diluted to 1:100 in blocking buffer and incubated overnight at 4°C. Secondary antibodies were diluted to 1:500 in the same buffer and incubated for 1 h at room temperature. After both primary and secondary antibody incubation steps, slides were washed three times for 10 min each wash in PBS with 0.05% Tween 20 (Sigma-Aldrich). After the immunostaining procedure, sections were mounted with Fluoroshield™ mounting medium with DAPI (F6057, Sigma-Aldrich^®^).

### Microscopy

A stereoscope (Nikon SMZ1500) with an attached camera (Nikon digital sight DS-Fi1) was used to take top view fluorescent images of whole heads.

Confocal microscopy was performed on a Leica Microsystems CMS TCS SP5 DM16000. Image sequences were reconstructed using Fiji (ImageJ) analysis software.

## Supplementary Material


