# Supplementary material for: Cranial suture lineage and contributions to repair of the mouse skull
Source: Development. 2024 Feb 12;151(3):dev202116. doi: 10.1242/dev.202116 (PMC10911112; doi:10.1242/dev.202116)
Supplement: Supplementary file 1 [file develop-151-202116_review_history.pdf]

Cranial suture lineage and contributions to repair of the mouse skull

Daniel Doro, Annie Liu, Jia Shang Lau, Arun Kumar Rajendran, Christopher Healy, Marko Krstic, Agamemnon E Grigoriadis, Sachiko Iseki and Karen J Liu
DOI: 10.1242/dev.202116

Editor: Patrick Tam

Review timeline

Original submission:	27 June 2023
Editorial decision:	28 July 2023
First revision received:	18 December 2023
Accepted:	8 January 2024

Original submission

First decision letter

MS ID#: DEVELOP/2023/202116

MS TITLE: The role of embryonic origins and suture proximity in repair of cranial bone

AUTHORS: Daniel Doro, Annie Liu, Jia Shang Lau, Arun Kumar Rajendran, Christopher Healy, Agamemnon E Grigoriadis, Sachiko Iseki, and Karen J Liu

I have now received all the referees' reports on the above manuscript, and have reached a decision. The referees' comments are appended below, or you can access them online: please go to BenchPressand click on the 'Manuscripts with Decisions' queue in the Author Area.

As you will see, the referees express considerable interest in your work, but have some significant criticisms and recommend a substantial revision of your manuscript before we can consider publication. If you are able to revise the manuscript along the lines suggested, which may involve further experiments, I will be happy receive a revised version of the manuscript. Your revised paper will be re-reviewed by one or more of the original referees, and acceptance of your manuscript will depend on your addressing satisfactorily the reviewers' major concerns. Please also note that Development will normally permit only one round of major revision.

If it would be helpful, you are welcome to contact us to discuss your revision in greater detail. Please send us a point-by-point response indicating your plans for addressing the referees' comments, and we will look over this and provide further guidance.

Please attend to all of the reviewers' comments and ensure that you clearly highlight all changes made in the revised manuscript. Please avoid using 'Tracked changes' in Word files as these are lost in PDF conversion. I should be grateful if you would also provide a point-by-point response detailing how you have dealt with the points raised by the reviewers in the 'Response to Reviewers' box. If you do not agree with any of their criticisms or suggestions please explain clearly why this is so.

Reviewer 1

Advance summary and potential significance to field

This study by Doro and coworkers investigates how embryonic origin and suture proximity correlates with ability to heal subcritical calvarial defects, and that the predominant cell type for defect

© 2024. Published by The Company of Biologists under the terms of the Creative Commons Attribution License (https://creativecommons.org/licenses/by/4.0/).

repair are neural crest-derived cells independent of the initial embryonic origin. The authors address an important gap of knowledge and show that anterior located coronal defects heal significantly better than posterior defects. They use lineage tracing to find that ability for defect repair correlates with the presence of neural crest-derived cells, and that neural crest-derived cells is the predominant cell type that contributes to defect repair. They also show that defects in the parietal region heal better in proximity to sutures.

Comments for the author

This is a nice study that addresses two not necessarily linked observations: 1) the influence of embryonic origin of cranial bones. A series of studies focusing on embryonic stages established the borders between neural crest and mesoderm domains including sutures. 2) the correlation of bone healing capacity to proximity to sutures. It is commonly assumed that the embryonic pattern of cell distribution is retained in adult mice. However, limited information available (PMID: 18228258) disputes this. Neural crest contribution via EMT from neural crest-derived dura mater cells to parietal bones is readily observed in mesoderm-derived parietal bones. The importance of suture proximity is more difficult to assess. Defect repair requires osteoblast precursors. Such precursor cells are found in sutures. The potential contribution from dura mater or their presence as bone-lining cells/correlation with active local bone remodeling are not investigated. Furthermore, the contribution of neural crest cells to repair of the various bones is only shown after but not prior to injury. Indeed, the authors show that defect repair involves recruitment of Gli1+ cells, a source of stem cells found in sutures, but also abundant in sutures and bone-lining cells.
Still, this is an overall carefully prepared study that adds important information to our understanding of the embryonic origin of cranial bones in young adult mice and their contribution to defect repair. However, the conclusion that neural crest-derived cells from sutures are responsible for defect repair is not directly supported. The authors show that all defects have substantial neural crest contribution at 1 week, while it appears that this relative contribution does not correlate with ability to heal (Fig. 4).

Specific comments:
Title is somewhat misleading, as suture proximity only plays a role in mesoderm-derived bones.
Some details on microCT are missing: voxel size for reconstruction and actual resolution of scans.
Immunofluorescence: details are missing, incl. antibody source
Confirm that cryosections were stored at room temperature
Details on mouse numbers, numbers of defects per mouse are missing. Also relative healing efficiencies within individual mice might provide useful information.
When referring to lineage-traced cells, make sure to indicate this consistently.
Refrain using e.g. Gli1+ cells when referring to Gli1+-derived cells.
It is unclear how many defects per made per mouse, and if this affected the outcome.
Fig. 2: It is not clear why the interparietal bone is shown as neural crest-derived.
Statements on the relative contribution of Axin2+-derived cells as a subset of Gli1+-derived cells is lacking experimental support. This could be addressed using reciprocal RNAscope paired with lineage tracing.
Fig. 4: Indicating the sites of defect in panels B-E would be helpful. Is panel F based on panels B-E? Ensure that all graphical representation faithfully capture the data presented or discuss if these are an average presentation of several (define number) of sections.
It is not clear if differential removal of the periosteum as part of the surgical procedure could have affected healing outcomes.
To test if local lining cells, dura mater-derived or suture cells contribute to defect repair, the authors could use cell dyes (DiI or similar) and label specific cell locations followed by fate analysis.
Consider that the mechanical environment of the anterior (rostral to coronal suture) vs posterior (caudal to coronal suture) sutures is quite different due to several factors including overall cranial anatomy/dimensions and the underlying brain (Marghoub 2018, PMID: 29243252).
The authors might consider to add in vitro competitive ossification assays starting with mixed mesodermal/neural crest cells and assess relative contribution to bone nodules.

Minor comments:
L115: correct: minimum critical distance -> maximum critical distance.
L129: correct: Cells that do not express lacking Wnt1::Cre

© 2024. Published by The Company of Biologists under the terms of the Creative Commons Attribution License (https://creativecommons.org/licenses/by/4.0/).

Reviewer 2

Advance summary and potential significance to field

This study provides some new information regarding the embryonic origins of cranial sutures in mouse as well as an indication that cranial neural crest cells and their derivatives contribute to wound healing of cranial vault bones.
Specifically, the authors combine calvarial wounding experiments with genetically labelled mouse models to determine whether suture proximity affects the efficiency of cranial repair under the assumption that if the wound is closer to CNC-derived sutural cells, the efficiency of cranial repair will be increased. Using genetic lineage labelling, the authors identify specific contributions of neural crest cells and associated skeletal stem cells of neural crest origin - apparently from the sutural stem cell niche - during repair of calvarial wounds. However, the lack of a mesoderm marker reduces the potential significance of the work.

Comments for the author

The initial and most prominent studies of neural crest/mesoderm derivation for sutures (e.g., Jiang PMID: 11784098 and Yoshida PMID: 18617001) use lacZ staining with Wnt1Cre and Mesp1Cre lines. By using fluorescent reporters, the authors of this paper have avoided problems common to these previous studies of suture derivation in which different ages, section planes, and locations within sutures often show varying quality of staining, sometimes appearing as inconsistencies between areas that are stained and leaving interpretation to the experience of the authors, the reviewers, and the readers. Fluorescent reporters avoid some of this, however, the lack of the use of a complementary labeling approach to mark mesoderm cells means that we can only be sure of the origin of GFP marked cells but not of the origin of cells marked with mTOM. Another thing I wonder about is whether some of the cells that remain unlabeled might mean that the cre simply isn't active in all cells, rather than the unrecombined cells are coming from a different origin. Adding a mesodermal marker to the study would help.

Wound Healing
The authors have clearly shown that the position of the defect determines the efficiency of wound healing but have interpreted that their findings show that the position of the defect determines the availability of neural crest-derived progenitors, and propose that neural crest cells are a key element in the repair of calvarial defects. I agree with the interpretation but do not find enough evidence to support this as fact (that their results are due to the proximity of suture dwelling neural crest cells) for problems outlined above and below regarding derivation of cells, especially lack of a mesoderm marker.

Suture derivation
The authors state on page 4 that “the embryonic origins of the cranial sutures are debatable, as they rely on early observations that lacked resolution.”
Sutures are surely dynamic systems. Deckelbaum PMID: 22395741 showed that the neural crest is excluded from the coronal suture mesenchyme, but that mesoderm cells do stray into the neural crest of the frontal bone. For the sagittal suture, the two parietal bones are mesoderm-derived, but the suture mesenchyme is thought to be neural crest. In lacZ stains with wnt1cre mice the sagittal suture mesenchyme looks like a tongue of mesenchyme extending from the neural crest between the mesoderm-derived parietal. The lambdoid suture mesenchyme appears to be totally mesoderm-derived, but the interparietal bone itself has that central domain of neural crest. The patterns are indeed complex -and puzzling- but are more understandable when we consider that during early head development the cells that will become suture cells are dragged along with the surface surface of the forming dura as the frontal lobes of the brain expand during growth. Studies of suture cells derivation and change over time are further elucidated by information provided by Gagan et al 2007 PMID: 18228258 who proposed that at postnatal day 10, neural crest cells are found throughout the intrafrontal suture and give rise to fibroblast-like mesenchymal cells in the sutures, as well as chondrocytes, osteoblasts, and osteocytes in developing bones. These authors also found that by postnatal day 20, the neural crest cells are restricted into discrete pockets of cells, particularly in the dura mater and bone marrow and that most of the cell population by postnatal day 20 appears to derive from an invasion of paraxial mesoderm into the tissues initially

© 2024. Published by The Company of Biologists under the terms of the Creative Commons Attribution License (https://creativecommons.org/licenses/by/4.0/).

formed entirely from neural crest-derived cells. All of this is to say that the cell populations that reside in the sutures are probably dynamic and it is going to require many different methodologies with data from pre and postnatal mice to understand these processes and the distribution of neural crest and mesoderm in the sutures. The data presented in this paper are a valuable contribution to that growing body of knowledge.

Detailed comments:
Page 4, lines 98-104: what is described here should be in the Methods section under subcritical defects

Page 4 lines 127-130 I think I understand this sentence but it is either missing words or has an inserted phrase that is not needed.

Page 5 lines 136-138 here it is stated that the "coronal section at a more anterior part of the sagittal suture shows complete absence of mesoderm (Figure 2B, 2C-E), contrasting a more posterior section which shows very few neural crest cells with abundant mesoderm." However, you cannot show a complete lack of mesoderm without a mesoderm marker.

Page 5 lines 140-141 Due to the lack of a mesoderm marker, this is not a neural crest-mesoderm boundary but instead a neural crest border.

Reviewer 3

Advance summary and potential significance to field

The research report “The role of embryonic origins and suture proximity in repair of cranial bone” by Doro et al. focuses on a novel point of view on the embryonic origins of cranial sutures in a mouse model. Furthermore, this study uses extensive genetic lineage labelling techniques combined with analysis of calvarial injury repair. This research report is concise and provides interesting new concepts. However, it could benefit from exploring further cellular and molecular mechanisms that would enrich the presented conclusions. Several important pieces of data that are necessary to draw the proposed conclusion are missing.

Comments for the author

Major points:

1. From Figure 3, the authors concluded that “all wounds had Gli1+ cells, regardless the proximity to the suture”. The authors rightly note that “quantitative assessment of the cell content in the wounds might reveal a correlation of distance vs. cell contribution.” Could the authors provide such quantitative assessment and other markers/or potential changes in signaling pathways and/or gene expression so that the cellular and molecular mechanism is expanded in this study? Moreover, the quotation above suggests that although mid-parietal defects are substantially more distant to the sutures than the other defects, they are still supplied with suture-derived osteoprogenitors, revealing the reparative capability of the suture mesenchyme in relation to distant defects. What is the cell fate of Gli1+ cells in the individual defects? Could the authors provide cell proliferation and differentiation analysis?
a. In the Results and Discussion section, the detailed description of Fig. 3B-H is missing.
b. Fig. 3G appears to have stronger expression of Gli1+ cells than the others. However, the amount of healing is the smallest. Could the authors give more detailed explanation relating these results?
2. Can the authors expand on the discussion related to the role of dura in their wound model? How would the contribution of the dura be distinguished in their model?
3. Fig. 1D provides extensive analysis of progressive healing after 1, 2, and 4 weeks visualized in a single graph. Could the authors show the skull scans after 4 weeks for individual defect positions?
4. The authors claimed that “the pericoronal parietal defect shows significantly more repair than the mid-pariental”, however, Fig. 1D showed that there is no significant difference between the pericoronal parietal and mid-parietal bone. The authors will need to discuss this?

© 2024. Published by The Company of Biologists under the terms of the Creative Commons Attribution License (https://creativecommons.org/licenses/by/4.0/).

5. In Fig. 4, GFP- cells should be stained with DAPI. In some instances, the yellow arrows point to black spaces (Fig. 4 j', k'). Could the quality of these images be improved? Overall, the imaging quality in Fig. 4 could be better, so the individual cells in the sutures and the bone can be distinguished.
6. The schematic drawing in Fig. 1A could benefit from labeling the individual sutures that the authors use in later descriptions. Even though the positions of the defects are named in the figure legend, the labeling in the figure would make it easier to orient the reader.
7. In Fig. 2O, is there an ectopic red signal on the right side?

Minor comments:

- The summary statement provided in lines 20-24 exceeds the allowance of 15 – 30 words.
- Some figure legends are missing N numbers (e.g., Figure 2, Figure 3, and Figure 4).
- There are two "that"s on Page 4, line 112-113.

First revision

Response to Reviewer Comments

MS ID#: DEVELOP/2023/202116
We thank the reviewers for their supportive critiques. We have responded to these comments with additional experiments and changes to the organisation of the manuscript, which we believe greatly improves our paper.
We include a point-by-point response. We also highlight the following major changes:

1) **Title:** We have changed the title to "Cranial suture lineage and contributions to repair of the mouse skull", which better reflects our findings.
2) **Addition of a new experiment**: we use *Mesp1::cre* lineage labelling to assess the mesodermal contributions to the postnatal adult skull. These are now shown in a substantially expanded Figure (new Figure 1), which allows us to assess these lineage contributions in parallel with the original transgenic *Wnt1::cre* mice.
3) **Reorganisation of the manuscript**, which we believe better suits the story. In brief, we now open with neural crest and mesodermal labelling of the skull.
    a. Figure 1: Lineage tracing of neural crest and mesoderm in the adult skull
    b. Figure 2: This figure now integrates localised wound healing (original Figure 1) and the *Wnt1::cre*-derived contributions to healing wounds (original Figure 4).
    c. Figure 3: *Gli1::creER*^{T2} *or Axin2::creER* lineage labelling (original Figure 2O-Z).
    d. Figure 4: Contributions of Gli1+-derived contributions to skull repair (original Fig 3).

Reviewer 1

Advance Summary and Potential Significance to Field:

This study by Doro and coworkers investigates how embryonic origin and suture proximity correlates with ability to heal subcritical calvarial defects, and that the predominant cell type for defect repair are neural crest-derived cells independent of the initial embryonic origin. The authors address an important gap of knowledge and show that anterior located coronal defects heal significantly better than posterior defects. They use lineage tracing to find that ability for defect repair correlates with the presence of neural crest-derived cells, and that neural crest-derived cells is the predominant cell type that contributes to defect repair. They also show that defects in the parietal region heal better in proximity to sutures.

Comments for the Author:

This is a nice study that addresses two not necessarily linked observations: 1) the influence of embryonic origin of cranial bones. A series of studies focusing on embryonic stages established the borders between neural crest and mesoderm domains including sutures. 2) the correlation of

© 2024. Published by The Company of Biologists under the terms of the Creative Commons Attribution License (https://creativecommons.org/licenses/by/4.0/).

bone healing capacity to proximity to sutures. It is commonly assumed that the embryonic pattern of cell distribution is retained in adult mice. However, limited information available (PMID: 18228258) disputes this. Neural crest contribution via EMT from neural crest-derived dura mater cells to parietal bones is readily observed in mesoderm-derived parietal bones. The importance of suture proximity is more difficult to assess. Defect repair requires osteoblast precursors. Such precursor cells are found in sutures. The potential contribution from dura mater or their presence as bone-lining cells/correlation with active local bone remodelling are not investigated. Furthermore, the contribution of neural crest cells to repair of the various bones is only shown after but not prior to injury. Indeed, the authors show that defect repair involves recruitment of Gli1+ cells, a source of stem cells found in sutures, but also abundant in sutures and bone-lining cells. Still, this is an overall carefully prepared study that adds important information to our understanding of the embryonic origin of cranial bones in young adult mice and their contribution to defect repair. However, the conclusion that neural crest-derived cells from sutures are responsible for defect repair is not directly supported. The authors show that all defects have substantial neural crest contribution at 1 week, while it appears that this relative contribution does not correlate with ability to heal (Fig. 4).

This reviewer raises several important points, which we agree with. We thank the reviewer for highlighting these points and have now included the following:

- The reviewer suggests our data does not discount the possibility that other tissues (e.g. dura mater) contribute to healing. We agree that this is a possibility; however, our data and others show that the suture and the sutural mesenchyme clearly have osteogenic capacity and respond rapidly to wounding. We have added the following text to the introduction:

Lines 54-58: "The healing capacity in the skull appears to rely on the sutural mesenchyme, which has recently proposed to act as a stem cell niche[4]. In response to wounding, the skeletal mesenchyme rapidly undergoes proliferation, with sutural cells expanding toward the wound. Several groups have demonstrated that ablation of these resident skeletal stem cells blocks the healing capacity of the skull[4-6]."

- To address limitations of our experiments, and potential roles of the *dura mater*, we have added the following:

Lines 220-234: "Our data suggest that the embryonic origins of the cranial bone may predict the outcome of defect repair due to the neural crest composition of the intervening sutures and the presence of suture-residing osteoprogenitors relative to the calvarial wound site. However, it is important to note that our observations are based on several critical assumptions. First, the genetic approaches used in our studies, while powerful, cannot exclude the possibility that these transgenic lines are not reactivated later in development, or subject to unknown transcriptional influences, or are simply not Cre-responsive. Second, it is important to note that meninges, notably the dura mater, are also neural crest derived[17,27,28]. Indeed, we and others have demonstrated that the dura mater has osteogenic capacity[29,30], and when co-cultured with parietal cells are capable of nucleating osteogenesis[8]. Finally, the composition of the sutural mesenchyme is surely dynamic, as the biological requirements change from embryogenesis to adult aging. Cell migration and mixing in adult life has been reported to occur in the sutures and in the meninges[27,31]. Furthermore, the meninges, which are comprised of neural crest cells during development, seem later to be invaded by mesodermal derivatives, leaving the remaining neural crest cells to act as resident stem cells in later life."

Specific comments:

Title is somewhat misleading, as suture proximity only plays a role in mesoderm-derived bones.

- **New title:** Cranial suture lineage and contributions to repair of the mouse skull

Some details on microCT are missing: voxel size for reconstruction and actual resolution of scans.

- This information has been added to the methods section (lines 290-295)..

Immunofluorescence: details are missing, incl. antibody source

© 2024. Published by The Company of Biologists under the terms of the Creative Commons Attribution License (https://creativecommons.org/licenses/by/4.0/).

- This information has been added to the methods section (lines 310-318).

Confirm that cryosections were stored at room temperature

- Information was fixed on the methods section (line 307).

Details on mouse numbers, numbers of defects per mouse are missing.

- We have included n-numbers in figure legends and added details to materials and methods.

Also, relative healing efficiencies within individual mice might provide useful information.

- Quantification of healing at experimental end-point is now in Figure 2D. We agree that relative healing efficiencies will be useful; unfortunately, we currently cannot track healing efficiencies during the lifetime of the animal.

When referring to lineage-traced cells, make sure to indicate this consistently. Refrain using e.g. Gli1+ cells when referring to Gli1+-derived cells.

- Fixed.

It is unclear how many defects per made per mouse, and if this affected the outcome.

- Information was added on the methods section (lines 274-278).

It is not clear why the interparietal bone is shown as neural crest-derived.

- Yoshida et al., have previously used *Wnt1::cre* and *Mesp1::cre Rosa*^{lacZ} reporters to demonstrate that the medial portion of the interparietal is neural crest derived. Our observations are consistent with those reports. We have amended the schematics in new Figure 1 to reflect this.

Statements on the relative contribution of Axin2+-derived cells as a subset of Gli1+-derived cells is lacking experimental support. This could be addressed using reciprocal RNA scope paired with lineage tracing.

- We thank the reviewer for this suggestion and agree that it would be interesting to perform *Axin2* mRNA expression analysis on the *Gli1*+-derived cells. Unfortunately, we do not have the capacity to perform reciprocal RNAscope/lineage tracing experiments at this time. Both Wilk et al., and Maruyama et al., include experiments supporting this hypothesis. We now include a comment stating:
  Lines 203-204: "Further assessment of *Axin2* mRNA expression on the *Gli1*+-derived cells would be necessary to definitively define these subpopulations of skeletal stem cells."

Fig. 4: Indicating the sites of defect in panels B-E would be helpful. Is panel F based on panels B-E?

- Figure 4 is now part of Figure 2. The sites of the defects in Fig 2F-I (previously 4B-E) are the same as those depicted in Figure 2E (previous 4A). We have omitted original Fig 4F to save space.

Ensure that all graphical representation faithfully capture the data presented or discuss if these are an average presentation of several (define number) of sections.

- We have confirmed our schematics and corrected any inaccuracies. Some of the graphical representations have been removed to accommodate new data. For all data shown we examined at least 3 sections from relevant regions in at least 3 animals.

It is not clear if differential removal of the periosteum as part of the surgical procedure could have

© 2024. Published by The Company of Biologists under the terms of the Creative Commons Attribution License (https://creativecommons.org/licenses/by/4.0/).

affected healing outcomes.

- The periosteal layer is removed with the help of a cotton bud, followed by 1mm defects with a dental drill, carefully avoiding the underlying dura mater. While it is possible that there is variable removal of the periosteum, we do not think this is the case. As the removal procedure was the same for all wounds in all animals (n=6/group), any variation should be internally controlled.

To test if local lining cells, dura mater-derived or suture cells contribute to defect repair, the authors could use cell dyes (DiI or similar) and label specific cell locations followed by fate analysis.

- We agree that these would be good experiments. Unfortunately, we do not have the resources to do these new wounding experiments. We note this in our revised conclusions.

Consider that the mechanical environment of the anterior (rostral to coronal suture) vs posterior (caudal to coronal suture) sutures is quite different due to several factors including overall cranial anatomy/dimensions and the underlying brain (Marghoub 2018, PMID: 29243252).

- We agree and have added the following comment in the conclusions:

  Line 235-242: "In the future, it will be important to track more localised cell contributions such as dura mater-derived cells or the adjacent periosteal cells. It will also be important to assess the environment unique to the frontal versus parietal bones: for example, Marghoub and colleagues have demonstrated distinct mechanical forces in the anterior versus the posterior sutures, due to the differences in bone size, anatomy and underlying brain morphology[32]. Altogether, while we cannot definitively state that availability of neural crest cells is the key element, we can nevertheless conclude that lineage identity and spatial positioning are both important in relation to the healing of calvarial defects."

The authors might consider to add in vitro competitive ossification assays starting with mixed mesodermal/neural crest cells and assess relative contribution to bone nodules.

- Thank you for this comment. Our group has previously performed and published these kinds of experiments. We now note this in the introduction (lines 64-67) and have added the reference.

  Lines 64-67: "In contrast, parietal osteoblasts rarely generate osteogenic nodules[8]; however, osteogenesis could be restored by co-culturing with neural crest-derived cells, either from frontal bones, or from the dura mater[8]."

Minor comments:
L115: correct: minimum critical distance -> maximum critical distance. Fixed.
L129: correct: Cells that do not express lacking Wnt1::Cre Fixed.

Reviewer 2

Advance Summary and Potential Significance to Field:

This study provides some new information regarding the embryonic origins of cranial sutures in mouse as well as an indication that cranial neural crest cells and their derivatives contribute to wound healing of cranial vault ones. Specifically, the authors combine calvarial wounding experiments with genetically labelled mouse models to determine whether suture proximity affects the efficiency of cranial repair under the assumption that if the wound is closer to CNC-derived sutural cells, the efficiency of cranial repair will be increased. Using genetic lineage labelling, the authors identify specific contributions of neural crest cells and associated skeletal stem cells of neural crest origin -apparently from the sutural stem cell niche - during repair of calvarial wounds. However, the lack of a mesoderm marker reduces the potential significance of

© 2024. Published by The Company of Biologists under the terms of the Creative Commons Attribution License (https://creativecommons.org/licenses/by/4.0/).

the work.

- We thank the reviewer for their support and agree that addition of a mesodermal marker would improve this work. We have now added *Mesp1::cre* lineage labelling of the adult skull (new Figure 1, lines 129-141) which showed that the mesodermal domain in the sutures indeed comprises the lambdoid and the posterior part of the sagittal suture, while the other sutures appear non-mesodermal. Given the dual origin of the cranial sutures clearly defined with the experiments in Figure 1, there may be a mixed contribution for local repair. Due to limited resources and time we are unfortunately unable to perform wounding experiments on the *Mesp1::cre* labelled animals. Nevertheless, we believe that our new experiments substantially add to the significance of our work.

Comments for the Author:

The initial and most prominent studies of neural crest/mesoderm derivation for sutures (e.g., Jiang PMID: 11784098 and Yoshida PMID: 18617001) use lacZ staining with Wnt1Cre and Mesp1Cre lines. By using fluorescent reporters, the authors of this paper have avoided problems common to these previous studies of suture derivation in which different ages, section planes, and locations within sutures often show varying quality of staining, sometimes appearing as inconsistencies between areas that are stained and leaving interpretation to the experience of the authors, the reviewers, and the readers. Fluorescent reporters avoid some of this, however, the lack of the use of a complementary labeling approach to mark mesoderm cells means that we can only be sure of the origin of GFP marked cells but not of the origin of cells marked with mTOM.

- We agree and have added *Mesp1::cre* lineage tracing as discussed above.

Another thing I wonder about is whether some of the cells that remain unlabelled might mean that the cre simply isn't active in all cells, rather than the unrecombined cells are coming from a different origin.

- This is entirely possible and is now noted in the conclusion.
  Lines 223-226: "First, the genetic approaches used in our studies, while powerful, cannot exclude the possibility that these transgenic lines are not reactivated later in development, or subject to unknown transcriptional influences, or are simply not Cre-responsive."

Wound Healing: The authors have clearly shown that the position of the defect determines the efficiency of wound healing but have interpreted that their findings show that the position of the defect determines the availability of neural crest-derived progenitors, and propose that neural crest cells are a key element in the repair of calvarial defects. I agree with the interpretation but do not find enough evidence to support this as fact (that their results are due to the proximity of suture dwelling neural crest cells) for problems outlined above and below regarding derivation of cells, especially lack of a mesoderm marker.

- Both reviewers agree on this point. We too agree, and have changed the title, and edited the manuscript to reflect this. We conclude:
  Lines 239-242: "Altogether, while we cannot definitively state that availability of neural crest cells is the key element, we can nevertheless conclude that lineage identity and spatial positioning are both important in relation to the healing of calvarial defects."

Suture derivation: The authors state on page 4 that "the embryonic origins of the cranial sutures are debatable, as they rely on early observations that lacked resolution." Sutures are surely dynamic systems. Deckelbaum PMID: 22395741 showed that the neural crest is excluded from the coronal suture mesenchyme, but that mesoderm cells do stray into the neural crest of the frontal bone. For the sagittal suture, the two parietal bones are mesoderm-derived, but the suture mesenchyme is thought to be neural crest. In lacZ stains with wnt1cre mice the sagittal suture mesenchyme looks like a tongue of mesenchyme extending from the neural crest between the mesoderm-derived parietal. The lambdoid suture mesenchyme appears to be totally mesoderm-

© 2024. Published by The Company of Biologists under the terms of the Creative Commons Attribution License (https://creativecommons.org/licenses/by/4.0/).

derived, but the interparietal bone itself has that central domain of neural crest. The patterns are indeed complex -and puzzling- but are more understandable when we consider that during early head development the cells that will become suture cells are dragged along with the surface surface of the forming dura as the frontal lobes of the brain expand during growth. Studies of suture cells derivation and change over time are further elucidated by information provided by Gagan et al 2007 PMID: 18228258 who proposed that at postnatal day 10, neural crest cells are found throughout the intrafrontal suture and give rise to fibroblast-like mesenchymal cells in the sutures, as well as chondrocytes, osteoblasts, and osteocytes in developing bones. These authors also found that by postnatal day 20, the neural crest cells are restricted into discrete pockets of cells, particularly in the dura mater and bone marrow and that most of the cell population by postnatal day 20 appears to derive from an invasion of paraxial mesoderm into the tissues initially formed entirely from neural crest-derived cells. All of this is to say that the cell populations that reside in the sutures are probably dynamic and it is going to require many different methodologies with data from pre and postnatal mice to understand these processes and the distribution of neural crest and mesoderm in the sutures. The data presented in this paper are a valuable contribution to that growing body of knowledge.

- As these important points were also raised by Reviewer 1, we have now amended our conclusion (lines 220-242) to take these into account.

Detailed comments:

Page 4, lines 98-104: what is described here should be in the Methods section under subcritical defects.

- Fixed

Page 4 lines 127-130 I think I understand this sentence but it is either missing words or has an inserted phrase that is not needed.

- Fixed!

Page 5 lines 136-138 here it is stated that the "coronal section at a more anterior part of the sagittal suture shows complete absence of mesoderm (Figure 2B, 2C-E), contrasting a more posterior section which shows very few neural crest cells with abundant mesoderm." However, you cannot show a complete lack of mesoderm without a mesoderm marker.

- Agreed, and we have amended the conclusion to reflect these limitations of our study.

Page 5 lines 140-141 Due to the lack of a mesoderm marker, this is not a neural crest-mesoderm boundary but instead a neural crest border.

- We agree and have added *Mesp1::cre* lineage tracing (Figure 1). Please see our detailed response regarding potential mesodermal contributions.

Reviewer 3

Advance Summary and Potential Significance to Field:

The research report "The role of embryonic origins and suture proximity in repair of cranial bone" by Doro et al. focuses on a novel point of view on the embryonic origins of cranial sutures in a mouse model. Furthermore, this study uses extensive genetic lineage labelling techniques combined with analysis of calvarial injury repair. This research report is concise and provides interesting new concepts. However, it could benefit from exploring further cellular and molecular mechanisms that would enrich the presented conclusions. Several important pieces of data that are necessary to draw the proposed conclusion are missing.

Comments for the Author:

Major points:

© 2024. Published by The Company of Biologists under the terms of the Creative Commons Attribution License (https://creativecommons.org/licenses/by/4.0/).

1. From Figure 3, the authors concluded that "all wounds had Gli1+ cells, regardless the proximity to the suture". The authors rightly note that "quantitative assessment of the cell content in the wounds might reveal a correlation of distance vs. cell contribution." Could the authors provide such quantitative assessment and other markers/or potential changes in signaling pathways and/or gene expression so that the cellular and molecular mechanism is expanded in this study? Moreover, the quotation above suggests that although mid-parietal defects are substantially more distant to the sutures than the other defects, they are still supplied with suture-derived osteoprogenitors, revealing the reparative capability of the suture mesenchyme in relation to distant defects. What is the cell fate of Gli1+ cells in the individual defects? Could the authors provide cell proliferation and differentiation analysis?

- These are all important and interesting questions! Unfortunately, we do not have the resources to pursue these questions at this time.

  Line 215-217: "In the future, a quantitative assessment of the signalling events and cell content will be important reveal the mechanistic links between distance, cell identity and healing."

a. In the Results and Discussion section, the detailed description of Fig. 3B-H is missing.

- Figure 3 is now Figure 4 and the description has been expanded. Lines 205-217.

b. Fig. 3G appears to have stronger expression of Gli1+ cells than the others. However, the amount of healing is the smallest. Could the authors give more detailed explanation relating these results?

- This is now Figure 4G. We have gone back to the raw data and reprocessed the images, which now see comparable.

2. Can the authors expand on the discussion related to the role of dura in their wound model? How would the contribution of the dura be distinguished in their model?

- As above, we have included further discussion of the dura mater.

3. Fig. 1D provides extensive analysis of progressive healing after 1, 2, and 4 weeks visualized in a single graph. Could the authors show the skull scans after 4 weeks for individual defect positions?

- This Figure is now part of Figure 2 and does indeed show 4 weeks after wounding.

4. The authors claimed that "the pericoronal parietal defect shows significantly more repair than the mid-parietal", however, Fig. 1D showed that there is no significant difference between the pericoronal parietal and mid-parietal bone. The authors will need to discuss this.

- This statement is an error. What we meant was that parietal defects performed near the sutures are not significantly different from frontal defects, whereas parietal defects in the middle of the bone heal less. This is now stated as "whereas the peri-coronal parietal defect healing comparable to the frontal bones" (line 154-155).

5. In Fig. 4, GFP- cells should be stained with DAPI. In some instances, the yellow arrows point toblack spaces (Fig. 4 j', k'). Could the quality of these images be improved? Overall, the imaging quality in Fig. 4 could be better, so the individual cells in the sutures and the bone can be distinguished.

- We have submitted higher resolution images and hope these are clearer.

6. The schematic drawing in Fig. 1A could benefit from labeling the individual sutures that the

© 2024. Published by The Company of Biologists under the terms of the Creative Commons Attribution License (https://creativecommons.org/licenses/by/4.0/).

authors use in later descriptions. Even though the positions of the defects are named in the figure legend, the labeling in the figure would make it easier to orient the reader.

- New labels were added to individual sutures.

7. In Fig. 2O, is there an ectopic red signal on the right side?

- Yes there is. This autofluorescence comes from opaque tissue left attached to the sample enhanced by the angle of the specimen in relation to the light source. This information was clarified in the corresponding figure legend.
  Lines 466-467: *Red to the right side is autofluorescence of opaque tissue left in the sample.

Minor comments:

•The summary statement provided in lines 20-24 exceeds the allowance of 15 - 30 words.

- Statement has been shortened to 30 words.

•Some figure legends are missing N numbers (e.g., Figure 2, Figure 3, and Figure 4).

- These have been fixed both in the legends, and in the methods.

•There are two “that”s on Page 4, line 112-113.

- Fixed.

Second decision letter

MS ID#: DEVELOP/2023/202116

MS TITLE: Cranial suture lineage and contributions to repair of the mouse skull

AUTHORS: Daniel Doro, Annie Liu, Jia Shang Lau, Arun Kumar Rajendran, Christopher Healy, Marko Krstic, Agamemnon E Grigoriadis, Sachiko Iseki, and Karen J Liu

ARTICLE TYPE: Research Report

I am happy to tell you that your manuscript has been accepted for publication in Development, pending our standard ethics checks.

Reviewer 1

Advance summary and potential significance to field

This study by Doro and coworkers investigates how embryonic origin and suture proximity correlates with ability to heal subcritical calvarial defects, and that the predominant cell type for defect repair are neural crest-derived cells independent of the initial embryonic origin.
The authors address an important gap of knowledge and show that anterior located coronal defects heal significantly better than posterior defects. They use lineage tracing to find that ability for defect repair correlates with the presence of neural crest-derived cells, and that neural crest-derived cells is the predominant cell type that contributes to defect repair. They also show that defects in the parietal region heal better in proximity to sutures. They also investigated the contribution of Mesp1+ mesenchymal cells.

Comments for the author

© 2024. Published by The Company of Biologists under the terms of the Creative Commons Attribution License (https://creativecommons.org/licenses/by/4.0/).

I thank the authors for diligently addressing the concerns of the reviews and adding novel data using the Mesp1Cre lineage tracing. This certainly strengthens the manuscript. As it stands, it provides important information on the origin of cranial cells in sutures and during bone defect repair. This article will become a point of reference for many craniofacial researchers.

I am sastisfied with the response, the reorganization of the manuscript and inclusion of novel data.

There is one minor points still to be addressed:
Line 157: whereas the pericoronal parietal defect healing comparable to the frontal bones (Figure 2D). This sentence is incomplete.

Reviewer 3

Advance summary and potential significance to field

All concerns have been addressed. the manuscript has improved following this revision.

Comments for the author

none

© 2024. Published by The Company of Biologists under the terms of the Creative Commons Attribution License (https://creativecommons.org/licenses/by/4.0/).
